# Heterogeneity and event dependence in the analysis of sickness absence

**DOI:** 10.1186/1471-2288-13-114

**Published:** 2013-09-16

**Authors:** Isabel Torá-Rocamora, David Gimeno, George Delclos, Fernando G Benavides, Rafael Manzanera, Josefina Jardí, Constança Alberti, Yutaka Yasui, José Miguel Martínez

**Affiliations:** 1 Department of Experimental and Health Sciences, Center for Research in Occupational Health (CiSAL), Universitat Pompeu Fabra (UPF), C/Doctor Aiguader 88, Barcelona 08003, Spain; 2 CIBER in Epidemiology and Public Health, Barcelona, Spain; 3 Southwest Center for Occupational and Environmental Health, Division of Epidemiology, Human Genetics and Environmental Sciences, The University of Texas School of Public Health, San Antonio Campus, San Antonio, TX, USA; 4 Southwest Center for Occupational and Environmental Health, Division of Epidemiology, Human Genetics and Environmental Sciences, The University of Texas School of Public Health, Houston Campus, Houston, TX, USA; 5 Institut Català d’Avaluacions Mèdiques i Sanitàries (ICAMS), Departament de Salut, Generalitat de Catalunya, Barcelona, Spain; 6 Department of Public Health Sciences, School of Public Health, University of Alberta, Edmonton, AB, Canada

**Keywords:** Sickness absence, Survival analysis, Conditional frailty model, Poisson regression, Mental disorders, Neoplasms

## Abstract

**Background:**

Sickness absence (SA) is an important social, economic and public health issue. Identifying and understanding the determinants, whether biological, regulatory or, health services-related, of variability in SA duration is essential for better management of SA. The conditional frailty model (CFM) is useful when repeated SA events occur within the same individual, as it allows simultaneous analysis of event dependence and heterogeneity due to unknown, unmeasured, or unmeasurable factors. However, its use may encounter computational limitations when applied to very large data sets, as may frequently occur in the analysis of SA duration.

**Methods:**

To overcome the computational issue, we propose a Poisson-based conditional frailty model (CFPM) for repeated SA events that accounts for both event dependence and heterogeneity. To demonstrate the usefulness of the model proposed in the SA duration context, we used data from all non-work-related SA episodes that occurred in Catalonia (Spain) in 2007, initiated by either a diagnosis of neoplasm or mental and behavioral disorders.

**Results:**

As expected, the CFPM results were very similar to those of the CFM for both diagnosis groups. The CPU time for the CFPM was substantially shorter than the CFM.

**Conclusions:**

The CFPM is an suitable alternative to the CFM in survival analysis with recurrent events, especially with large databases.

## Background

Sickness absence (SA) is a complex phenomenon with great economic and social impact, and is considered a major occupational and public health issue [1-3]. SA is defined as a temporary situation in which a worker is unable to perform his/her usual work, either because of illness or injury [4]. The duration of SA affects the individual worker’s quality of life, and have a great impact in his/her family, employer and society overall [5]. Knowing what factors are associated with how long a sickness absence episode lasts is of great importance in trying to reduce the SA duration. Sickness absence duration has been examined using a number of statistical techniques, most frequently survival analysis techniques [6-8]. Generally, survival studies analyze the time until the occurrence of a certain event of interest (e.g., death) [9]. However, in the context of sickness absence, some individuals may be more prone to experience multiple events, whether due to new illnesses or injuries, or recurrence of the same event. Repeated events can create within-subject correlation in event times [8,10-12], arising from two sources: 1) event dependence; and 2) heterogeneity across individuals [11]. Event dependence occurs when the risk of a particular event depends on events previously experienced, whereas heterogeneity occurs when some individuals have a higher or lower risk of experiencing the events due to unknown, unmeasured or unmeasurable factors. Consequently, analytical approaches to modeling of sickness absence duration should take into account both event dependence and heterogeneity to avoid obtaining biased estimates of the parameters of interest [11,12].

The conditional frailty model (CFM) proposed by Box-Steffensmeier and De Boef [11], which can be viewed as an extension of the Cox model, simultaneously captures event dependence and heterogeneity [11], and has been used previously in political sciences research [12]. The computational applicability of the CFM maybe limited when dealing with very large datasets such a sickness absence registries, numbering hundreds of thousands or millions of individuals and/or episodes. For example, in Catalonia for the year 2007, the Catalan Institute of Medical and Health Evaluations (ICAMS, by its Spanish acronym) recorded 800,464 sickness episodes in 580,959 persons. It is well established that Poisson regression is a possible alternative to Cox regression [13,14]. Specifically, when a Cox model is confronted with computational limitations in analyzing large databases, a Poisson regression model maybe a reasonable alternative [15].

The goal of this paper is to propose a Poisson-based conditional frailty model (CFPM) that accounts for event dependence and heterogeneity for a large data analysis of sickness absence.

## Methods

In the first section we will introduce the CFM and explain the proposed CFPM. In the following section we will explain the methods used to empirically compare the CFM and CFPM.

### Conditional frailty and conditional frailty Poisson models

#### The conditional frailty model

The CFM models the dependence of events and heterogeneity by stratifying the baseline hazard function by event order and incorporating random effects for individuals, respectively. The formulation of the model is in gap time so that time at risk is reset after each event. Let *λ*_*ik*_(*t*) the hazard of *k*-th event occurring in the *i*-th individual, the CFM is defined as

$$\lambda_{\mathrm{ik}}\left(t\right)=\lambda_{0k}\left(t-t_{k-1}\right)e^{X_{\mathrm{ik}}^{T}\beta +\omega_{i}}$$

where t_k-1_ is the time of occurrence of (k-1)^th^ event, *λ*_0k_(t - t_*k* - 1_) is the baseline hazard rate for the *k*-th event, *β* is the vector of parameters associated with covariates X and *ω*_*i*_ is the random effect or “frailty” of the *i*-th individual that follows a gamma distribution. Considering right-censored failures, the parameters are interpreted as the log hazard ratio estimates associated with covariates for an event since the previous event, due to the gap time data structure incorporated in (t - t_*k* - 1_). More details about the CFM can be found in Box-Steffensmeier *et al.*[11,12].

#### The conditional frailty Poisson model

The CFPM considers $\lambda_{\mathrm{ik}}^{*}\left(t\right)$ to be the hazard of *k*-th event at time *t* occurring in the *i*-th individual, as

$$\lambda_{\mathrm{ik}}^{*}\left(t\right)=\lambda_{0k}^{*}\left(t-t_{k-1}\right)e^{X_{\mathrm{ik}}^{T}\beta +\omega_{i}}$$

Following the piecewise exponential model formulation [16], the baseline hazard for the *k*-th event is defined as

$$\lambda_{0k}^{*}\left(t\right)=\sum_{j=1}^{J}\lambda_{\mathrm{jk}}^{*}\cdot I_{\left\{t\in (\tau_{j},\tau_{j+1}]\right\}}$$

with divisions of time scale into (*τ*_1_, *τ*_2_], (*τ*_2_, *τ*_3_], …, (*τ*_J_, *τ*_*∞*_] which are J non-zero, nonoverlapping intervals, with *τ*_1_ = 0. The model captures event dependence (i.e., the dependence of the risk of a subsequent event on the occurrences of previous events) by allowing the baseline hazard to vary by event orders using an index “*k*” for the baseline hazard $\lambda_{0k}^{*}$ for the *k*^th^ event. The heterogeneity is controlled by including an ω_i_ random effect for the *i-*th individual. We consider a gamma distribution for the random effect.

Let *n*_*jik*_ and *d*_*jik*_ denote the time at risk and a covariate indicator of an event (*d*_*jik*_ = 1) or non-event (*d*_*jik*_ = 0), in the *j*-th time interval, for *i-*th individual and *k-*th event. The proposed Poisson regression model assumes a Poisson distribution on *d*_*jik*_|*ω*_*i*_ with the following log-linear mean,

$$\text{log}\left(E\left[d_{\text{jik}}|\omega_{i}\right]\right)=\text{log}\left(\lambda_{\mathrm{ik}}^{*}\left(t\right)\right)+\text{log}\left(n_{\mathrm{jik}}\right)$$

Note that the observed duration of SA (“time at risk under observation”) is include as an offset term in the Poisson model which starts on the day of SA certification and ends on the day the worker returns to work or the day the worker’s SA status becomes unknown (e.g., due to retirement, death, emigration), whichever is earlier.

### Empirical comparison between conditional frailty models

#### Description of the data

The CFM and CFPM were compared empirically using data from all episodes of non work-related SA that occurred in Catalonia (Spain) in 2007 (n = 800,464). Specifically, we assessed the influence of certain covariates of interest on SA duration, where the end of the episode of SA is considered the event of interest. A same individual may suffer more than one SA during the study period and therefore SAs are repeatable events.

The data were recorded through the Integrated Management System for Sickness Absence (SIGIT*,* by its Spanish acronym) at the ICAMS, a computerized registry and connected to all physicians in Catalonia responsible for certifying SA episodes.

For each episode, the diagnosis at case closure was available, coded according to the International Classification of Diseases, 10th Edition (ICD-10). We separately analyzed two large ICD-10 diagnosis groups selected to reflect frequent SA diagnoses (mental and behavioral disorders, codes F00-F99) and SA diagnoses with typically long duration times (neoplasms, codes C00-D48). Mental and behavioural disorders accounted for 3,268,075 days from 59,647 episodes in 53,238 individuals with a median duration of 10 days (25th percentile, 25 days; 75th percentile, 67 days); and neoplasms accounted for 516,676 days from 7,431 episodes in 6,975 individuals with median duration of 11 days (25th percentile, 28 days; 75^th^ percentile, 80 days). Approximately 10% of individuals had repeated events. For neoplasms, repeated events occur in 5% of individuals. Problems with convergence may emerge if there are too many event-order strata and/or a small number of episodes per stratum in both CFM [12] and CFPM. Therefore, we collapsed the event number so that any number of repeated episodes greater than 5 was set equal to 5.

Other covariates of interest were sex, age (16–28, 29–35, 36–45, >45 years), economic activity (11 branches), Catalonian health region, entity responsible for case management (National Institute of Social Security or a mutual insurance company), and employment status (salaried or self-employed).

#### Empirical comparison

We empirically compared the hazard ratio (HR) and 95% confidence intervals (95% CI) obtained by the CFM and the proposed CFPM. To define the baseline hazard function in the CFPM following the piecewise exponential model, we chopped time into 90-day-length non-overlapping.

To explore the source of correlation existing in the data and to better assess the proposed CFPM as a reliable alternative to the CFM, we also computed the HR and 95% CI, with models which: 1) only take into account the event dependence; or 2) only take into account for heterogeneity. The former models were based on a gap time conditional model (CM) [17] which takes into account the event dependence by stratifying the baseline hazard function according to event order [18]. The CM is similar to CFM but does not include the individual random effect term. We also ran a conditional Poisson model (CPM) with the same expression as the CFPM, but without the random effect term by individual. With respect to models that control only for heterogeneity we considered a frailty model (FM), which is similar to the CFM but without stratifying the baseline hazard functions by event order and controls for the heterogeneity by including random effects for individuals. Finally, we ran a Poisson model that takes into account only heterogeneity (FPM). The FPM presents a similar expression to the CFPM, but without the interaction between event order and the baseline hazard function.

Based on Box-Steffensmeier and De Boef [11] we hypothesized that when event dependence is strong, the event-dependence-only models (CM and CPM) should give estimates of the effects which are closed to the CFM, than models that do not control for the dependence of events (FM and FPM). Similarly, if heterogeneity is strong, the results of frailty models (FM and FPM) should be closer to the CFM than the models which only take into account dependence of events (CM and CPM). For both cases, i.e., regardless of the cause of correlation that predominates (event dependence or heterogeneity), we should expect that the estimates of CFPM will be closer to the CFM than the other models that only control for event dependence or only for heterogeneity. Thus, the comparison of the different models with the CFM serves to evaluate the suitability of CFPM when there is event dependence and/or heterogeneity.

The results between models were compared using the % relative bias (%RB) in point estimate and the % relative width difference in confidence interval (%RW), using the CFM as reference [15]. These measures are defined as

$$\%\;\mathrm{RB}=\left(\frac{HR_{\text{Other}}-HR_{\text{CFM}}}{HR_{\text{CFM}}}\right)\times 100$$

$$\%\;\mathrm{RW}=\left(\frac{\left(U_{\text{Other}}-L_{\text{Other}}\right)-\left(U_{\text{CFM}}-L_{\text{CFM}}\right)}{\left(U_{\text{CFM}}-L_{\text{CFM}}\right)}\right)\times 100$$

where HR_Other_ and HR_CFM_ are the hazard ratio under a specific model (CM, FM, CPM, FPM, CFPM) and the CFM, respectively, and U_Other_ and L_Other_ are the respective upper and lower confidence interval endpoints under a specific model, and U_CFM_ and L_CFM_ represent the upper and lower confidence interval endpoints for the CFM, respectively.

To compare the time for obtaining the parameter estimates from CFPM and CFM, their respective CPU time was measured on the Windows XP operating system on Intel® Core™2 CPU machine. The CFM and CFPM were fitted using R version 2.8.1 and Stata version 11, respectively. The Stata code for the CFPM, FPM and CPM is provided in the Additional file 1. Specifically we used the poisson command for the CPM, and the xtpoisson command for the CFPM and the FPM. Information about these commands can be found in the book written by Rabe-Hesketh S and Skrondal A [19].

This study was approved by the Parc de Salut Mar Clinical Research Ethical Committee of Barcelona, Spain (number 2011/4229/I).

## Results

Tables 1 and 2 summarize the HR estimates and 95% CIs, for the six models, adjusted for covariates, for mental or behavioural disorders and neoplasms, respectively.

**Table 1:** Hazard ratio and 95% confidence interval for selected covariates from episodes of non work-related sickness absence occurred in Catalonia (Spain) in 2007 for mental or behavioural disorders (n = 59,647)

		**CFPM**	**CPM**	**FPM**	**CM**	**FM**	**CFM**
		**HR (95% CI)**	**HR (95% CI)**	**HR (95% CI)**	**HR (95% CI)**	**HR (95% CI)**	**HR (95% CI)**
**Gender**							
	Male	1.00 (-)	1.00 (-)	1.00 (-)	1.00 (-)	1.00 (-)	1.00 (-)
	Female	0.91 (0.88-0.93)	0.93 (0.91-0.95)	0.90 (0.88-0.92)	0.93 (0.92-.95)	0.89 (0.87-0.92)	0.92 (0.90-0.94)
**Age at onset of absence episode (years)**							
	16 - 28	1.00 (-)	1.00 (-)	1.00 (-)	1.00 (-)	1.00 (-)	1.00 (-)
	29 - 35	0.84 (0.81-0.87)	0.86 (0.84-0.89)	0.84 (0.81-0.87)	0.88 (0.86-0.90)	0.82 (0.79-0.86)	0.85 (0.82-0.88)
	36 - 45	0.76 (0.73-0.78)	0.81 (0.78-0.83)	0.76 (0.73-0.78)	0.82 (0.80-0.84)	0.73 (0.71-0.76)	0.78 (0.75-0.80)
	> 45	0.63 (0.61-0.65)	0.70 (0.68-0.72)	0.63 (0.60-0.65)	0.72 (0.70-0.74)	0.59 (0.57-0.62)	0.66 (0.64-0.68)
**Health region**							
	Barcelona	1.00 (-)	1.00 (-)	1.00 (-)	1.00 (-)	1.00 (-)	1.00 (-)
	Lleida	1.05 (0.98-1.12)	1.05 (1.00-1.11)	1.04 (0.97-1.12)	1.04 (1.00-1.09)	1.04 (0.97-1.12)	1.05 (0.98-1.11)
	Camp de Tarragona	1.09 (1.04-1.15)	1.03 (0.98-1.07)	1.10 (1.05-1.16)	1.04 (1.00-1.08)	1.16 (1.09-1.23)	1.07 (1.02-1.12)
	Terres de l’Ebre	1.09 (0.99-1.21)	1.06 (0.98-1.15)	1.09 (0.99-1.20)	1.05 (0.97-1.13)	1.10 (0.98-1.23)	1.08 (0.98-1.18)
	Girona	1.01 (0.97-1.05)	1.01 (0.98-1.04)	1.01 (0.97-1.05)	1.01 (0.98-1.04)	1.00 (0.96-1.05)	1.01 (0.97-1.04)
	Catalunya Central	1.06 (1.01-1.11)	1.04 (1.00-1.08)	1.06 (1.01-1.11)	1.04 (1.00-1.07)	1.06 (1.01-1.12)	1.05 (1.01-1.10)
	Alt Pirineu i Aran	0.99 (0.79-1.25)	0.95 (0.78-1.16)	0.99 (0.78-1.25)	0.94 (0.79-1.13)	1.01 (0.78-1.31)	0.97 (0.79-1.19)
**Economic activity branch**							
	Agriculture, mining, fishing	1.00 (-)	1.00 (-)	1.00 (-)	1.00 (-)	1.00 (-)	1.00 (-)
	Manufacturing industry, energy production	1.04 (0.71-1.52)	0.99 (0.72-1.35)	1.05 (0.72-1.55)	1.01 (0.76-1.32)	1.09 (0.71-1.67)	1.03 (0.73-1.45)
	Construction	1.02 (0.70-1.49)	0.99 (0.72-1.36)	1.02 (0.69-1.50)	1.01 (0.76-1.33)	1.05 (0.68-1.51)	1.01 (0.72-1.43)
	Commercial/vehicles repair	0.89 (0.61-1.30)	0.88 (0.64-1.20)	0.89 (0.61-1.31)	0.91 (0.69-1.19)	0.91 (0.59-1.39)	0.89 (0.64-1.26)
	Hotel, restaurant businesses	0.88 (0.60-1.29)	0.87 (0.63-1.19)	0.88 (0.60-1.30)	0.90 (0.68-1.18)	0.90 (0.58-1.38)	0.89 (0.63-1.25)
	Transportation/communication	0.91 (0.62-1.34)	0.90 (0.66-1.24)	0.92 (0.63-1.36)	0.93 (0.70-1.22)	0.94 (0.61-1.44)	0.92 (0.65-1.30)
	Finance, real estate, services	0.92 (0.63-1.35)	0.91 (0.66-1.24)	0.93 (0.63-1.37)	0.93 (0.71-1.23)	0.95 (0.62-1.46)	0.93 (0.66-1.30)
	Government	0.90 (0.62-1.31)	0.89 (0.65-1.22)	0.91 (0.62-1.34)	0.92 (0.70-1.21)	0.92 (0.60-1.42)	0.91 (0.64-1.27)
	Health, education, other social activities	0.95 (0.65-1.39)	0.95 (0.69-1.30)	0.96 (0.65-1.41)	0.97 (0.73-1.27)	0.98 (0.64-1.50)	0.96 (0.68-1.35)
	Domestic housekeeping	0.92 (0.53-1.60)	0.94 (0.61-1.44)	0.91 (0.52-1.59)	0.96 (0.56-1.41)	0.93 (0.50-1.74)	0.94 (0.57-1.54)
	Extraterritorial agencies	0.69 (0.39-1.22)	0.72 (0.49-1.08)	0.69 (0.39-1.23)	0.77 (0.54-1.10)	0.68 (0.36-1.30)	0.73 (0.44-1.22)
**Social Security regime**							
	Salaried	1.00 (-)	1.00 (-)	1.00 (-)	1.00 (-)	1.00 (-)	1.00 (-)
	Self-employed	0.65 (0.61-0.69)	0.79 (0.76-0.82)	0.64 (0.60-0.68)	0.80 (0.78-0.83)	0.58 (0.54-0.62)	0.71 (0.67-0.75)
**Entity manage**							
	National Institute of Social Security	1.00 (-)	1.00 (-)	1.00 (-)	1.00 (-)	1.00 (-)	1.00 (-)
	Insurance company	1.14 (1.11-1.16)	1.11 (1.08-1.13)	1.14 (1.11-1.17)	1.09 (1.08-1.11)	1.15 (1.12-1.19)	1.12 (1.10-1.15)

*Abbreviations*: *HR* hazard ratio, *95% CI* confidence intervals at 95%, *CFPM* conditional frailty Poisson model, *CPM* conditional poisson model, *FPM* frailty poisson model, *CM* conditional model, *FM* frailty model, *CFM* conditional frailty model.

**Table 2:** Hazard ratio and 95% confidence interval for selected covariates from episodes of non work-related sickness absence occurred in Catalonia (Spain) in 2007 for neoplasms (n = 7,431)

	**CFPM**	**CPM**	**FPM**	**CM**	**FM**	**CFM**
	**HR (95% CI)**	**HR (95% CI)**	**HR (95% CI)**	**HR(95%CI)**	**HR (95% CI)**	**HR (95% CI)**
**Gender**						
Male	1.00 (-)	1.00 (-)	1.00 (-)	1.00 (-)	1.00 (-)	1.00 (-)
Female	0.92 (0.85-0.99)	0.95 (0.89-1.01)	0.92 (0.86-0.99)	0.95 (0.90-1.00)	0.89 (0.81-0.97)	0.91 (0.84-0.98)
**Age at onset of absence episode (years)**						
16 – 28	1.00 (-)	1.00 (-)	1.00 (-)	1.00 (-)	1.00 (-)	1.00 (-)
29 – 35	0.79 (0.69-0.90)	0.81 (0.70-0.93)	0.80 (0.70-0.92)	0.82 (0.73-0.93)	0.76 (0.64-0.89)	0.76 (0.66-0.87)
36 – 45	0.55 (0.49-0.62)	0.62 (0.55-0.70)	0.56 (0.50-0.64)	0.66 (0.59-0.73)	0.51 (0.44-0.59)	0.53 (0.46-0.60)
> 45	0.31 (0.28-0.35)	0.41 (0.37-0.47)	0.32 (0.28-0.36)	0.45 (0.41-0.50)	0.26 (0.22-0.30)	0.29 (0.26-0.33)
**Health region**						
Barcelona	1.00 (-)	1.00 (-)	1.00 (-)	1.00 (-)	1.00 (-)	1.00 (-)
Lleida	1.09 (0.91-1.30)	1.14 (0.99-1.31)	1.12 (0.94-1.34)	1.12 (1.00-1.26)	1.08 (0.87-1.33)	1.09 (0.90-1.30)
Camp de Tarragona	1.19 (1.03-1.37)	1.13 (0.99-1.28)	1.22 (1.06-1.41)	1.13 (1.01-1.26)	1.35 (1.13-1.61)	1.22 (1.05-1.41)
Terres de l’Ebre	1.19 (0.96-1.47)	1.26 (1.08-1.48)	1.17 (0.94-1.46)	1.22 (1.06-1.39)	1.14 (0.88-1.48)	1.17 (0.93-1.46)
Girona	0.88 (0.79-0.97)	0.92 (0.84-1.00)	0.88 (0.79-0.98)	0.92 (0.85-0.99)	0.86 (0.76-0.97)	0.86 (0.77-0.96)
Catalunya Central	0.94 (0.82-1.08)	0.95 (0.85-1.06)	0.95 (0.83-1.09)	0.96 (0.87-1.06)	0.95 (0.81-1.12)	0.95 (0.82-1.09)
Alt Pirineu i Aran	0.86 (0.56-1.31)	0.96 (0.72-1.27)	0.84 (0.54-1.29)	0.96 (0.75-1.24)	0.79 (0.47-1.31)	0.84 (0.54-1.30)
**Economic activity branch**						
Agriculture, mining, fishing	1.00 (-)	1.00 (-)	1.00 (-)	1.00 (-)	1.00 (-)	1.00 (-)
Manufacturing industry, energy production	0.67 (0.34-1.30)	0.70 (0.38-1.27)	0.68 (0.35-1.35)	0.73 (0.44-1.21)	0.70 (0.32-1.51)	0.68 (0.34-1.35)
Construction	0.55 (0.28-1.08)	0.62 (0.34-1.14)	0.57 (0.29-1.13)	0.66 (0.40-1.09)	0.55 (0.25-1.20)	0.55 (0.28-1.11)
Commercial/vehicles repair	0.59 (0.30-1.15)	0.64 (0.35-1.17)	0.61 (0.31-1.21)	0.68 (0.41-1.12)	0.58 (0.27-1.26)	0.59 (0.30-1.18)
Hotel, restaurant businesses	0.53 (0.27-1.05)	0.57 (0.31-1.06)	0.54 (0.27-1.08)	0.61 (0.36-1.02)	0.52 (0.23-1.14)	0.53 (0.26-1.07)
Transportation/communication	0.68 (0.34-1.33)	0.70 (0.38-1.30)	0.70 (0.35-1.40)	0.74 (0.44-1.24)	0.69 (0.31-1.51)	0.68 (0.34-1.37)
Finance, real estate, services	0.59 (0.30-1.15)	0.64 (0.35-1.18)	0.60 (0.30-1.19)	0.68 (0.41-1.12)	0.57 (0.26-1.25)	0.59 (0.30-1.18)
Government	0.59 (0.30-1.15)	0.63 (0.34-1.15)	0.60 (0.30-1.19)	0.67 (0.40-1.11)	0.59 (0.27-1.29)	0.59 (0.30-1.18)
Health, education, other social activities	0.61 (0.31-1.19)	0.65 (0.36-1.20)	0.62 (0.31-1.23)	0.69 (0.42-1.15)	0.62 (0.29-1.35)	0.61 (0.31-1.22)
Domestic housekeeping	0.49 (0.20-1.23)	0.60 (0.30-1.21)	0.50 (0.19-1.27)	0.65 (0.36-1.19)	0.44 (0.15-1.35)	0.49 (0.19-1.27)
Extraterritorial agencies	0.28 (0.10-0.77)	0.41 (0.20-0.80)	0.28 (0.10-0.79)	0.45 (0.25-0.81)	0.23 (0.07-0.77)	0.28 (0.10-0.79)
**Social Security regime**						
Salaried	1.00 (-)	1.00 (-)	1.00 (-)	1.00 (-)	1.00 (-)	1.00 (-)
Self-employed	0.73 (0.65-0.82)	0.84 (0.78-0.91)	0.70 (0.62-0.79)	0.86 (0.80-0.92)	0.60 (0.52-0.69)	0.71 (0.62-0.80)
**Entity manage**						
National Institute of Social Security	1.00 (-)	1.00 (-)	1.00 (-)	1.00 (-)	1.00 (-)	1.00 (-)
Insurance company	1.05 (0.98-1.12)	1.03 (0.97-1.09)	1.05 (0.98-1.13)	1.02 (0.97-1.08)	1.06 (0.98-1.15)	1.05 (0.97-1.12)

*Abbreviations*: *HR* hazard ratio, *95 CI%* confidence intervals at 95%, *CFPM* conditional frailty poisson model, *CPM* conditional poisson model, *FPM* frailty poisson model, *CM* conditional model, *FM* frailty model, *CFM* conditional frailty model.

The six models we considered showed associations that were in the same direction (for a specific group of a covariate the HR were above 1 (or below 1) in the six models). Although the associations for all six models were in the same direction, there were differences in the magnitude of HR across the models. The CFPM results were very similar to those of the CFM for both diagnosis groups (Tables 3 and 4).

**Table 3:** Percentage of relative bias in point estimates and percentage of relative width differences in confidence intervals for selected covariates for episodes of non work-related sickness absence due to mental or behavioural disorders occurring in Catalonia (Spain) in 2007

		**CFPM**		**CPM**		**FPM**		**CM**		**FM**	
		**%RB**	**%RW**	**%RB**	**%RW**	**%RB**	**%RW**	**%RB**	**%RW**	**%RB**	**%RW**
**Gender**											
	Male	-	-	-	-	-	-	-	-	-	-
	Female	-1.1	25.0	1.1	0.0	-2.2	0.0	1.1	-25.0	-3.3	25.0
**Age at onset of absence episode (years)**											
	16 - 28	-	-	-	-	-	-	-	-	-	-
	29 - 35	-1.2	0.0	1.2	-16.7	-1.2	0.0	3.5	-33.3	-3.5	16.7
	36 - 45	-2.6	0.0	3.9	0.0	-2.6	0.0	5.1	-20.0	-6.4	0.0
	> 45	-4.6	0.0	6.1	0.0	-4.6	25.0	9.1	0.0	-10.6	25.0
**Health region**											
	Barcelona	-	-	-	-	-	-	-	-	-	-
	Lleida	0.0	7.7	0.0	-15.4	-1.0	15.4	-1.0	-30.8	-1.0	15.4
	Camp de Tarragona	1.9	10.0	-3.7	-10.0	2.8	10.0	-2.8	-20.0	8.4	40.0
	Terres de l’Ebre	0.9	10.0	-1.9	-15.0	0.9	5.0	-2.8	-20.0	1.9	25.0
	Girona	0.0	14.3	0.0	-14.3	0.0	14.3	0.0	-14.3	-1.0	28.6
	Catalunya	1.0	11.1	-1.0	-11.1	1.0	11.1	-1.0	-22.2	1.0	22.2
	Alt Pirineu	2.1	15.0	-2.1	-5.0	2.1	17.5	-3.1	-15.0	4.1	32.5
**Economic activity branch**											
	Agriculture, mining, fishing	-	-	-	-	-	-	-	-	-	-
	Manufacturing industry, energy production	1.0	12.5	-3.9	-12.5	1.9	15.3	-1.9	-22.2	5.8	33.3
	Construction	1.0	11.3	-2.0	-9.9	1.0	14.1	0.0	-19.7	4.0	31.0
	Commercial/vehicles repair	0.0	11.3	-1.1	-9.7	0.0	12.9	2.3	-19.4	2.3	29.0
	Hotel, restaurant businesses	-1.1	11.3	-2.3	-9.7	-1.1	12.9	1.1	-19.4	1.1	29.0
	Transportation/communication	-1.1	10.8	-2.2	-10.8	0.0	12.3	1.1	-20.0	2.2	27.7
	Finance, real estate, services	-1.1	12.5	-2.2	-9.4	0.0	15.6	0.0	-18.8	2.2	31.3
	Government	-1.1	9.5	-2.2	-9.5	0.0	14.3	1.1	-19.1	1.1	30.2
	Health, education, other social activities	-1.0	10.5	-1.0	-9.0	0.0	13.4	1.0	-19.4	2.1	28.4
	Domestic housekeeping	-2.1	10.3	0.0	-14.4	-3.2	10.3	2.1	-22.7	-1.1	27.8
	Extraterritorial agencies	-5.5	6.4	-1.4	-24.4	-5.5	7.7	5.5	-28.2	-6.9	20.5
**Social Security regime**											
	Under contract	-	-	-	-	-	-	-	-	-	-
	Self-employed	-8.5	0.0	11.3	-25.0	-9.9	0.0	12.7	-37.5	-18.3	0.0
**Entity manage**											
	National Institute of Social Security	-	-	-	-	-	-	-	-	-	-
	Insurance company	1.8	0.0	-0.9	0.0	1.8	20.0	-2.7	-40.0	2.7	40.0

*Abbreviations*: *%RB* % relative bias, *%RW* % relative width, *CFPM* conditional frailty poisson model, *CPM* conditional poisson model, *FPM* frailty poisson model, *CM* conditional model, *FM* frailty model, *CFM* conditional frailty model.

**Table 4:** Percentage of relative bias in point estimates and percentage of relative width differences in confidence intervals for selected covariates for episodes of non work-related sickness absence due to neoplasms occurring in Catalonia (Spain) in 2007

		**CFPM**		**CPM**		**FPM**		**CM**		**FM**	
		**%RB**	**%RW**	**%RB**	**%RW**	**%RB**	**%RW**	**%RB**	**%RW**	**%RB**	**%RW**
**Gender**											
	Male	-	-	-	-	-	-	-	-	-	-
	Female	1.1	0.0	4.4	-14.3	1.1	-7.1	4.4	-28.6	-2.2	14.3
**Age at onset of absence episode (years)**											
	16 - 28	-	-	-	-	-	-	-	-	-	-
	29 - 35	4.0	0.0	6.6	9.5	5.3	4.8	7.9	-4.8	0.0	19.1
	36 - 45	3.8	-7.1	17.0	7.1	5.7	0.0	24.5	0.0	-3.8	7.1
	> 45	6.9	0.0	41.4	42.9	10.3	14.3	55.2	28.6	-10.3	14.3
**Health region**											
	Barcelona	-	-	-	-	-	-	-	-	-	-
	Lleida	0.0	-2.5	4.6	-20.0	2.8	0.0	2.8	-35.0	-0.9	15.0
	Camp de Tarragona	-2.5	-5.6	-7.4	-19.4	0.0	-2.8	-7.4	-30.6	10.7	33.3
	Terres de l’Ebre	1.7	-3.8	7.7	-24.5	0.0	-1.9	4.3	-37.7	-2.6	13.2
	Girona	2.3	-5.3	7.0	-15.8	2.3	0.0	7.0	-26.3	0.0	10.5
	Catalunya Central	-1.1	-3.7	0.0	-22.2	0.0	-3.7	1.1	-29.6	0.0	14.8
	Alt Pirineu I Aran	2.4	-1.3	14.3	-27.6	0.0	-1.3	14.3	-35.5	-6.0	10.5
**Economic activity branch**											
	Agriculture, mining, fishing	-	-	-	-	-	-	-	-	-	-
	Manufacturing industry, energy production	-1.5	-5.0	2.9	-11.9	0.0	-1.0	7.4	-23.8	2.9	17.8
	Construction	0.0	-3.6	12.7	-3.6	3.6	1.2	20.0	-16.9	0.0	14.5
	Commercial/vehicles repair	0.0	-3.4	8.5	-6.8	3.4	2.3	15.3	-19.3	-1.7	12.5
	Hotel and restaurant businesses	0.0	-3.7	7.6	-7.4	1.9	0.0	15.1	-18.5	-1.9	12.4
	Transportation/communication	0.0	-3.9	2.9	-10.7	2.9	1.9	8.8	-22.3	1.5	16.5
	Finance, real estate, services	0.0	-3.4	8.5	-5.7	1.7	1.1	15.3	-19.3	-3.4	12.5
	Government	0.0	-3.4	6.8	-8.0	1.7	1.1	13.6	-19.3	0.0	15.9
	Health, education, other social activities	0.0	-3.3	6.6	-7.7	1.6	1.1	13.1	-19.8	1.6	16.5
	Domestic housekeeping	0.0	-4.6	22.5	-15.7	2.0	0.0	32.7	-23.2	-10.2	10.2
	Extraterritorial agencies	0.0	-2.9	46.4	-13.0	0.0	0.0	60.7	-18.8	-17.9	1.5
**Social Security regime**											
	Under contract	-	-	-	-	-	-	-	-	-	-
	Self-employed	2.8	-5.6	18.3	-27.8	-1.4	-5.6	21.1	-33.3	-15.5	-5.6
**Entity manage**											
	National Institute of Social Security	-	-	-	-	-	-	-	-	-	-
	Insurance company	0.0	-6.7	-1.9	-20.0	0.0	0.0	-2.9	-26.7	1.0	13.3

*Abbreviations*: *%RB* % relative bias, *%RW* % relative width, *CFPM* conditional frailty poisson model, *CPM* conditional poisson model, *FPM* frailty poisson model, *CM* conditional model, *FM* frailty model, *CFM* conditional frailty model.

For neoplasms, the %RB for the CFPM ranged from 0% to 6.9% (absolute values), and the %RW from 0% to 7.1%. For the FPM and FM, these results were not as close to the CFM as the CFPM (10.3% in the %RB and 14.3% in the %RW for age > 45 in the FPM, and -17.9% in the %RB for extraterritorial agencies, and 33.3% in the %RW for Camp de Tarragona Health Region in the FM). The results for the CM and CPM were further apart from the CFM as compared to the FPM and FM, in some cases %RB reaching the 20%, 40% or, in the case of CM, 60% and%RW exceeding the 20%.

For mental or behavioural disorders, the CFPM, CPM and FPM behaved similar and were better than the CM and FM, and the CFPM behaved very closely to the CFM. In terms of %RW in general the CFPM presents the lowest percentages, but they can be up to 15%. In the case of CM and FM, the %RW can reach 30-40%.

The CPU time for the CFPM was much shorter than the CFM. Using R version 2.8.1. on the Windows XP operating system on Intel® Core™2 CPU machine, the CFM took 124,877.67 (2,081.30 minutes) and 647.53 seconds (10.80 minutes) CPU time for mental health disorders and neoplasm data analysis, respectively. Using Stata version 11, on the same operating system and hardware, the CFPM took 260.56 (4.34 minutes) and 35.77 (0.60 minutes) seconds for mental health disorders and neoplasm, respectively.

## Discussion

We proposed for the first time a Poisson-based conditional frailty model that accounts for both event dependence and heterogeneity. The CPU time required for the CFPM was substantially shorter than that for the CFM. In addition, as expected, the CFPM results were very similar to those of the CFM for both diagnosis groups.

The similarity of results between the CFM and CFPM, and the differences noted with models that do not include event dependence and/or heterogeneity reinforces the usefulness of the CFPM. In the case of neoplasms, the %RB for frailty models is closer to the CFM than for conditional models, suggesting that the dependence that dominates the data is heterogeneity. Conversely, in the case of mental health disorders, the %RB is smaller in the CM than that of the FM, indicating a greater influence of event dependence.

The choice of time intervals may influence the model fit result. The key issue is to sufficiently approximate the underlying hazard function over time by a set of piecewise-constant hazards in Poisson models. The shorter we make the time intervals of the piecewise-constant hazards the closer Poisson models get to Cox models. If data in each time interval become sparse by making the intervals shorter, however, parameter estimation becomes unstable, which in turn affect the estimation of the covariates’ effects of interest. As Michael Friedman pointed out “precise practical guidelines for choosing the number of intervals have not been formulated” [20]. Choosing different cut-points has a trade-off. It will be helpful to explore the form of the underlying hazard function and also assess the availability of data in each interval. In addition, performing a sensitivity analysis choosing different cut-points is useful for assessing changes in the parameters estimates of interest.

To avoid convergence problems we treated repeated episodes greater than 5 as equal to 5. The percentage of individuals with more than 5 repeated episodes for neoplasms is 0.52%, and for mental and behavioural disorders is 0.35%. Due to the very low percentages of individuals with more than 5 episodes, treating episodes greater than 5 as equal to 5 do not change the results.

A key advantage of the CFPM over the CFM is the reduction of computational time when analyzing large databases. This may be particularly important for institutions in countries where computers with high computational speed are not readily available. Currently, the CFM can only be run using R version 2.8.1. software [21]. The CFPM, though, can easily be run using other, statistical software such as Stata [22].

## Conclusions

In summary, assuming that within-subject correlation is a result of event dependence will result in biased estimates when, in fact, it is due to heterogeneity in the data. Conversely, assuming correlation in event times is due to heterogeneity will also result in biased inferences when, in fact, the source is event dependence [12]. For this reason, we recommend incorporating both sources of correlation when fitting a model. To achieve this, the CFPM is an attractive alternative to the CFM in survival analysis with recurrent events, especially with large databases, such as those that may exist for the analysis of sickness absence data.

## Abbreviations

SA: Sickness absence; ICAMS (by its Spanish acronym): Catalan Institute of Medical and Health Evaluations; CFM: Conditional frailty model; CFPM: Poisson-based conditional frailty model; SIGIT (by its Spanish acronym): Integrated management system for sickness absence; ICD-10: International classification of diseases 10th edition; HR: Hazard ratio; CM: Conditional model; CPM: Conditional poisson model; FM: Frailty model; FPM: Frailty poisson model; %RB: % Relative bias; %RW: % Relative width difference in confidence interval; CPU: Central processing unit.

## Competing interests

The authors declare that they have no competing interests.

## Authors’ contributions

ITR, YY, and JMM conceived the idea and worked on in the statistical and public health issues of the method, conducted the data analysis, and wrote the initial draft. DG, GD, and FGB gave critiques from public-health points of view and study design. JJ, RM, and CA provided discussion based on sickness absence. CA assisted the data analyses. All authors reviewed numerous drafts of the manuscript, are in agreement with the text and findings, and we have all approved this final version.

## Pre-publication history

The pre-publication history for this paper can be accessed here:

http://www.biomedcentral.com/1471-2288/13/114/prepub

## Supplementary Material

Additional file 1 The file includes the Stata syntax for Conditional Frailty Poisson Model (CFPM), Frailty Poisson Model (FPM) and Conditional Poisson Model (CPM). Click here for file
