# Supplementary material for: Heterogeneity and event dependence in the analysis of sickness absence
Source: BMC Med Res Methodol. 2013 Sep 16;13:114. doi: 10.1186/1471-2288-13-114 (PMC3852331; doi:10.1186/1471-2288-13-114)
Supplement: Additional file 1 — The file includes the Stata syntax for Conditional Frailty Poisson Model (CFPM), Frailty Poisson Model (FPM) and Conditional Poisson Model (CPM). [file 1471-2288-13-114-S1.doc]

*#######################################################################################

*#Stata syntax for Conditional Frailty Poisson Model (CFPM), Frailty Poisson Model (FPM) *#and Conditional Poisson Model (CPM) ##################################################

*#Variables definition#

*For illustration purposes only, we have considered one explanatory covariate (variable sex).

* id : id number

* duration : sickness absence duration in days

* event : event or censored time indicator with values 1 (event) and 0 (censored)

* episode : episode number with values 1 (episode #1), 2 (episode #2), 3 (episode

* #3), 4 (episode #4) and 5 (episode #>=5)

* sex : sex with values 1 (female) and 0 (male)

*#Split sickness absence duration every 90 days#

generate idp=_n

stset duration, failure(event==1) id(idp)

drop duration

stsplit duration, every(90)

generate spduration=_t-_t0

list id _t0 _t _d duration spduration if idp<=10,sepby(idp)

*#Dummy covariates for episode number#

generate I2=0

replace I2=1 if episode==2

generate I3=0

replace I3=1 if episode==3

generate I4=0

replace I4=1 if episode==4

generate I5=0

replace I5=1 if episode==5

*#Conditional Frailty Poisson Model (CFPM)#

xi:xtpoisson _d sex i._t0*I2 i._t0*I3 i._t0*I4 i._t0*I5, i(id) exposure(spduration) irr

*#Frailty Poisson Model (FPM)#

xi:xtpoisson _d sex i._t0, i(id) exposure(spduration) irr

*#Conditional Poisson Model (CPM)#

*#The option cluster() implies robust error variance#

xi:poisson _d sex i._t0*I2 i._t0*I3 i._t0*I4 i._t0*I5, cluster(id) exposure(spduration) irr
